# Calcined Eggshell as a P Reactive Media Filter—Batch Tests and Column Sorption Experiment

**DOI:** 10.1007/s11270-018-4068-7

**Published:** 2019-01-09

**Authors:** Agnieszka Bus, Agnieszka Karczmarczyk, Anna Baryła

**Affiliations:** Faculty of Civil and Environmental Engineering, Department of Environmental Improvement, Warsaw University of Life Sciences WULS – SGGW, Nowoursynowska 166, 02-787 Warsaw, Poland

**Keywords:** Calcined egg shells, Column experiment, Phosphorus retention, Pollution, Reactive materials

## Abstract

The goal of the study was to assess the sorption properties of calcined eggshells (CEs) as a P reactive media filter. The CEs were calcined in a temperature of 900 °C. A double stage test was performed: batch studies (kinetic and equilibrium) and small-scale column experiment. The estimation of optimal mass ratio of CEs for perspective usage was the additional benefit of column experiment. The short kinetic tests showed that 5 min of contact time with solution of initial concentration of 6.020 mgP-PO_4_ L^−1^ is enough to reduce the P-PO_4_ in 100%. The equilibrium studies were conducted with P-PO_4_ solution of 6.020 to 977.7 mg L^−1^ with contact time of 30 min. The obtained data was compensated by non-linear regression using the Marquardt algorithm in the Statgraphics Centurion XVI. The eggshell calcined characterized by high sorption capacity (*S*_max_ = 72.87 mg g^−1^) obtained from the Langmuir isotherm model with a good fit (96.77%). To choose the appropriate ratio of a sand filter to eggshells amendment, four small columns were constructed and fed with P-PO_4_ solution (*C*_in_ ≈ 5 mg L^−1^). The percentage mass (m/m) of CEs in the columns was 0.0 (the reference one); 1.0; 2.5; and 5.0. The unit sorption obtained during 95 days of column experiment was 10.668, 4.277, and 2.286 mg P-PO_4_ g^−1^ for 1.0, 2.5, and 5.0%, respectively. For practical implementation, the most recommended addition seems to be 1% of CEs. It corresponds, e.g., to the mass of 49 kg CEs for septic tank system.

## Introduction

Water and wastewater contaminated with phosphorous are still a significant environmental problem. Surplus of phosphorus (P) introduced into the environment may lead to deterioration of surface and ground water quality and soil. Water bodies’ eutrophication is the most significant effect of water pollution that causes not only environmental (Smith and Schindler 2009) but also economic costs (Withers et al. 2014).

One of the options of reducing P in environment is trapping when it moves through the landscape by reactive media filters (RMFs). There are many different materials proposed to be used as P reactive materials (RMs) which can be categorized as natural (e.g., dolomite, limestone, shell sand, shells, sands, serpentinite), industrial by-product (e.g., autoclaved aerated concrete, ashes, and slags), and man-made products (e.g., LECA, Filtralite P®). An important issue in the case of RMs is the cost of acquisition, transport, and availability. Since many years, RMs are used to reduce the P concentration from wastewaters as a support P reduce filter at on-site wastewater treatment plant (Vidal et al. 2018, Renman and Renman 2010) or additional filter materials for filling the bed of constructed wetland (Jóźwiakowski 2012, Jóźwiakowski et al. 2017).

One of new approach to P reduction from water and wastewaters is using waste materials and by-products. Chicken eggshells are world-wide available waste, which can be used as a P RMF. A global egg production in 2016 r. was estimated at 73.9 million metric tons (www.statista.com). Assuming that the eggshells represent 11% of total weight of egg (Carvalho et al. 2011), it gave 8.13 million metric tons of by-products. Most of eggshells by-product are disposed in landfills without any pretreatment. The other ways of applications are reused as a fertilizer, feed addictive (Carvalho et al. 2011), use a as hydroxyapatite sources in medicine (Pluta et al. 2017), and RM for removing: lead ion (Vijayaraghavan & Joshi 2013), copper ion (Vijayaraghavan et al. 2005), uranium and thorium (Ishikawa et al. 2002), and dyes such as methylene blue (Tsai et al. 2006). The eggshells are characterized by a porous nature and high, rather stable content of CaCO_3_, ranged from 94% (Carvalho et al. 2011, Köse and Kıvanç 2011) to 97% (Mezenner and Bensmaili 2009). That gives this material a great potential to remove P from aqueous solutions. The affinity to bind P may be increased by calcination process. The thermal treatment of eggshell at temperature of 900–1200 °C promotes the decomposition of CaCO_3_ to CaO (Pluta et al. 2017). Tsai et al. (2006) reported that BET surface area of eggshells increase from 1.053 to 1.845 m^2^ g^−1^ after the process of calcination at 1000 °C. Also, the increase of particle porosity was observed, from 0.0182 (raw eggshell) to 0.0510 g cm^−1^ (eggshell after calcination at 1000 °C). Thus, calcined eggshells are a potential world-wide available by-product and environmentally reasonable P RMF.

The novelty of the study is to use calcined eggshells (CEs) as a biosorbent for treatment artificial wastewater in a form of column experiment. Based on literature review, the usefulness of CEs to reduce phosphorus from aquatic solution was tested only during bath tests (Köse and Kıvanç 2011, Guo et al. 2017, Mezenner and Bensmaili 2009, Zhang et al. 2018). There were no attempts to test the CEs as P reactive media in a form of the filter.

The goal of the study was to assess the sorption properties of CEs as a P reactive media filter. A double stage test was performed: batch studies (kinetic and equilibrium) and small-scale column experiment. The estimation of optimal mass ratio of CEs for perspective usage was the additional benefit of column experiment.

## Material and Methods

### Material

Collected eggshells were rinsed in a distilled water to completely remove a residue of egg white and yolk. Also, the egg double membranes were manually removed to get clean, raw shells. The resultant eggshells were dried naturally and subsequently grounded (IKA A10) for a powder fraction. Finally, the obtained powder was calcined in the furnace (Nabertherm P330) at 900 °C for 3 h. The mineral composition of raw (Zhang et al. 2018) and CEs with SEM photography is presented in Table 1.

**Table 1:**
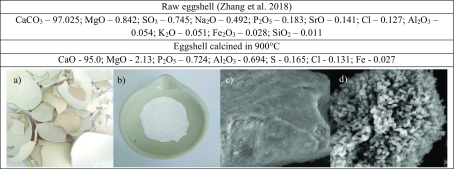
Main mineral composition [%] of raw and eggshell calcined in 900 °C with figures of (a) raw chicken eggshells, (b) eggshell powder before calcination, (c) SEM microphotography of raw eggshell powder, and (d) calcined in 900 °C (both at scale 20µm)

### Batch Tests

Varying concentrations of the artificial P solution prepared of KH_2_PO_4_ were used in all batch tests for assessing P sorption. The triplicate samples of material were mixed in an Erlenmeyer glass flask, each contained 0.5 g of material and 50 mL (10 g:1 L) of the various P solution added. The kinetic tests were performed at various contact times (5–60 min) and the constant solution concentration of 6.020 mg P-PO_4_ L^−1^. The sorption equilibrium tests were performed at various solution concentrations (6.020–977.7 mg P-PO_4_ L^−1^) and a constant time (30 min.). The pH was measured by Volcraft PH-212 m.

The P removal ratio *R* (%) was calculated based on the equation:

1 $$ R\ \left[\%\right]=\frac{C_0-{C}_e}{C_0}\bullet 100\% $$

where *C*_0_ and *C*_e_ are the initial and equilibrium P concentration (mg L^−1^).

The sorption capacity (*q*_e_) was calculated from the following equation:

2 $$ {q}_{\mathrm{e}}=\frac{\left({C}_0-{C}_{\mathrm{e}}\right)\bullet V}{m} $$

where *q*_e_ is sorption capacity (mg g^−1^); *V* is the volume of solution (L); *m* is the mass of material (g), and *C*_0_ and *C*_e_ are the initial and final (equilibrium) concentrations (mg L^−1^).

Description of the sorption process between solid phase and solution was made based on mathematical equations given by Langmuir (McKay 1996):

3 $$ {q}_{\mathrm{e}}=\frac{q_{\mathrm{max}}\bullet {K}_{\mathrm{L}}\bullet {C}_{\mathrm{e}}}{1+{K}_{\mathrm{L}}\bullet {C}_{\mathrm{e}}} $$

where *q*_e_ is sorption capacity (mg/g); *q*_max_ is maximum sorption capacity (mg g^−1^); *K*_L_ is Langmuir adsorption constant related to the affinity of binding sites (L/g); and *C*_e_ is equilibrium concentration (mg L^−1^)

and Freundlich equation (McKay 1996):

4 $$ {q}_{\mathrm{e}}={K}_{\mathrm{F}}\bullet {C}_{\mathrm{e}}^{\frac{1}{n}} $$

where *C*_e_ is equilibrium concentration (mg L^−1^); *q*_e_ is adsorption capacity (mg g^−1^); *K*_F_ is Freundlich constant related to the adsorption capacity (L g^−1^); and *n* is constant factor (−).

The Langmuir isotherm describes adsorption on homogenous surfaces while Freundlich isotherm assumes surface which is heterogeneous.

The essential features of the Langmuir isotherm may be expressed in terms of equilibrium parameter *R*_L_, which is a dimensionless constant referred to as separation factor or equilibrium parameter (Shabudeen et al. 2006):

5 $$ {R}_{\mathrm{L}}=\frac{1}{1+{K}_{\mathrm{L}\bullet {C}_0}} $$

where *C*_0_ is initial concentration and *K*_L_ is constant related to the energy of adsorption (the Langmuir constant). The values of *R*_L_ indicate the isotherm and adsorption nature to be either unfavorable if *R*_L_ > 1, linear if *R*_L_ = 1, favorable if 0 < *R*_L_ < 1, or irreversible if *R*_L_ = 0 (Shabudeen et al. 2006).

The P equilibrium concentrations were measured by flow injection analyses using FIAstar 500. All samples were double filtered: firstly by hard paper filter and secondly by syringe filter of 0.45 μm pore size. The obtained data was compensated by non-linear regression using the Marquardt algorithm in the Statgraphics Centurion XVI.

### Column Experiment

**Fig. 1:**
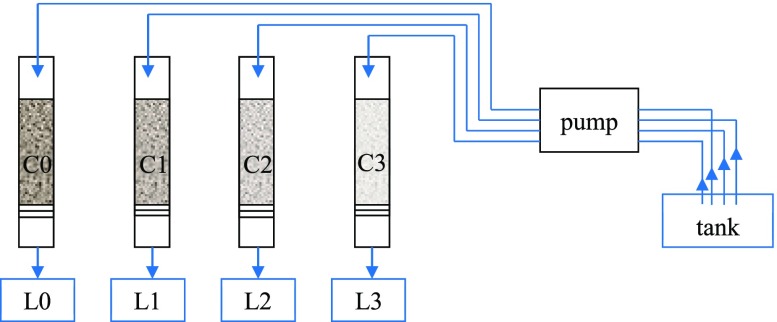
Setup of column experiment. C0, reference one; C1, 1.0% m/m of CEs; C2, 2.5% m/m of CEs; C3, 5% m/m of CEs; L0–L3, leakage from columns 0 to 3

Four small columns (fi = 14 mm, *h* = 250 mm) with bed height of 150 mm were filled with sand (as a reference filter) and mixture of sand and CEs (900 °C) of mass percentage (m/m): 1.0, 2.5, and 5.0 (Fig. 1, Table 2). The sand used in the experiment was sifted (0.05–2.0 mm) and washed out from phosphorus in demonized water. The columns were underlying by thick, double filtration fleece to prevent flush out the CEs amendments.

**Table 2:** Parameters of column experiment: C0–C03, columns 0–3; CE, calcined eggshell; HLR, hydraulic loading rate; TDS, total dissolved solids; EC, electronic conductivity; SD, standard deviation

	Column			
	C0 (reference)	C1	C2	C3
Mass of sand [g]	40	39.6	39.0	38.0
Mass of CE [g]	0.0	0.4 (1.0%)	1.0 (2.5%)	2.0 (5.0%)
Q average ± SD [mL min^−1^]	0.0218 ± 0.0091	0.0218 ± 0.0085	0.0216 ± 0.0060	0.0217 ± 0.0068
HLR [L m^−2^ day^−1^]	204	204	202	203
Initial (tank) concentrations				
	TDS [ppm]	EC [mS cm^−1^]	pH [−]	P-PO_4_ [mg L^−1^]
Average ± SD	411.50 ± 33.63	0.77 ± 0.09	7.63 ± 0.36	4.989 ± 0.619

The four-channel peristaltic pomp Lead Fluid® BT100S (Lead Fluid Technology Co., LTD.) was used to pump constantly the synthetic solution from tank to columns C0–C3. The flow was set to 0.1 rpm per 1 min; however, during the experiment, it was observed that pump channels do not work evenly and the SD value of flow from C0 to C3 equal 20.63 mL.

The filters were fed up by P-PO_4_ synthetic solution (prepared by KH_2_PO_4_) stored in a 5-L tank. Characterization of initial tank concentration is set in Table 2. The synthetic solution and column leaching were volume measured and examined for pH (Volcraft PH-212 m), electronic conductivity (EC) (Con110, Lovibond) and TDS (EZ-1) before filtration. After that, the samples were double filtered (hard paper and 0.45 μm syringe filter) and frozen until P-PO_4_ measurement by FIAstar 5000 flow injection analyzer (Foss) in a range 0.005–1.000 and 0.100–5.000 mgP-PO_4_ L^−1^.

The experiment lasted 95 days and during this time 42 samples were taken from each column. Statistical analysis for the measured values of all parameters was conducted using STATISTICA version 12. software produced by StatSoft (www.statsoft.com). Pearson’s coefficient was calculated for the parameters measured during small-scale column experiment with the level of significance set at *p* < 0.05.

## Results

### Kinetic and Sorption Equilibrium Tests

Based on short kinetic tests (5–60 min), the CEs are characterized by fast and rapid P-PO_4_ sorption (Table 3). As expected, the P-PO_4_ removal increased rapidly in a very short contact time and then achieved equilibrium. After 5 min of contact time, an entire load of P-PO_4_ was removed from the solution.

**Table 3:** Kinetic results of eggshells calcined in 900 °C; *C*_0_ = 6.020 mgP-PO_4_ L^−1^

Time [min]	5	15	30	60
Average ± SD				
Sorption [mg g^−1^]	0.602 ± 0.00	0.601 ± 0.01	0.602 ± 0.00	0.602 ± 0.00
Reduction [%]	100.0 ± 0.00	99.8 ± 0.01	100.0 ± 0.00	100.0 ± 0.00

Based on the kinetic tests, the contact time for equilibrium tests were set for 30 min.

**Fig. 2:**
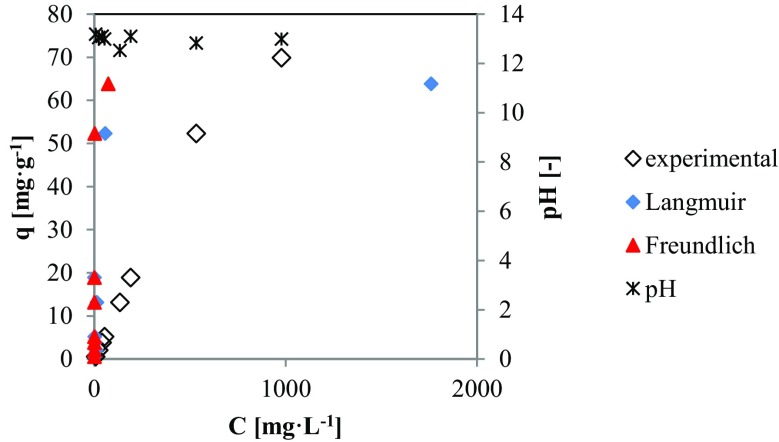
The pH and sorption properties for CEs heated in 900 °C

The experimental data obtained for CEs (Fig. 2) were fitted to the Langmuir and Freundlich adsorption isotherms. The data shows a good compliance with the Langmuir isotherm model (96.78%) and the regression coefficients for the plots were higher than for the Freundlich isotherm (Table 4). Maximal sorption capacity (*q*_max_) calculated according the Langmuir isotherm parameters equals 72.87 mgP-PO_4_ g^−1^. This value is close to that observed during sorption equilibrium tests (69.93 mgP-PO_4_ g^−1^). Because the Langmuir isotherm the best reflects the mechanism of P sorption, also the *R*_L_ factor obtained from the isotherm indicate that adsorption model is favorable and equals 0.66.

**Table 4:** The Langmuir and Freundlich isotherm parameters for P adsorption

Langmuir isotherm			Freundlich isotherm		
*K*_L_	*q*_max_	*R*^2^ [%]	*K*_F_	*n*	*R*^2^ [%]
*0.0855*	*72.87*	96.78	0.0466	0.1787	35.54

Values in italics are with a significance level at *p* < 0.05

The observed pH were highly alkaline and stable at all tested concentrations and ranged from 12.53 to 13.19 (Fig. 2).

### Column Experiment

**Fig. 3:**
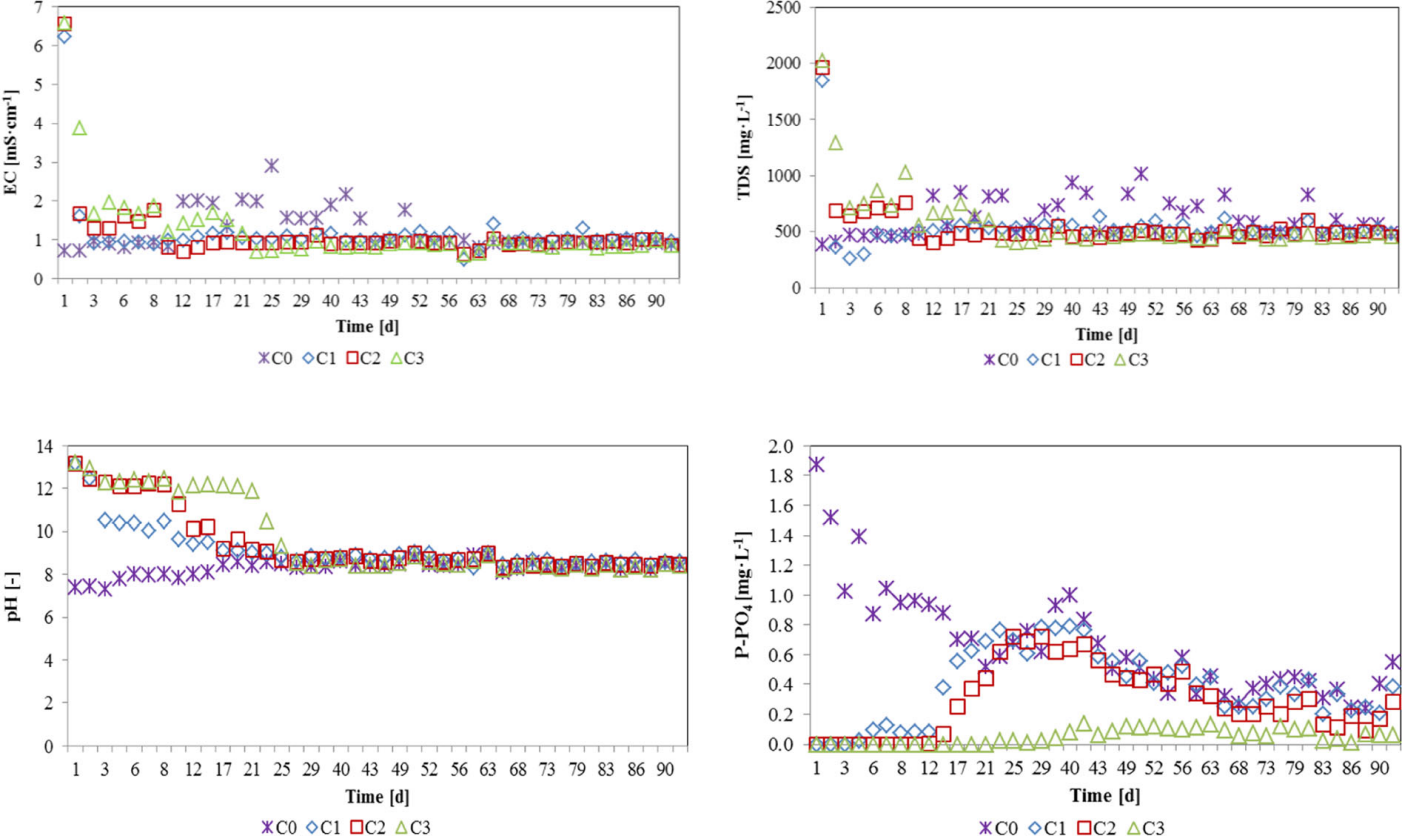
Evolution of electronic conductivity (EC), total dissolved solids (TDS), pH, and P-PO_4_ inlet concentration of C0–C3

Electronic conductivity (EC) and total dissolved solids (TDS) showed a very rapid decrease during the first days of operation. The highest values were observed over the first 2 days of experiment which is connected with washing out the Ca ions from the columns. After that, the leachates from C1 to C3 were stabilized (Fig. 3). This is well seen in significance Pearson coefficient (*R*^2^ > 94%) for C1–C3 (Tables 6, 7, and 8) and much more lower value (*R*^2^ = 49%) for C0 (Table 5).

**Table 5:** Pearson correlation coefficients between physical, chemical parameter leaching, and flow for C0

	Q	TDS	pH	EC	P-PO_4_	Average	SD
Q	1.000000					0.0218	0.0091
TDS	*− 0.343858*	1.000000				615.4762	162.9983
pH	*− 0.444265*	*0.464077*	1.000000			8.2890	0.3514
EC	− 0.303364	*0.495635*	0.285541	1.000000		1.2293	0.5138
P-PO_4_	*0.439959*	− 0.250893	*− 0.709815*	0.064637	1.000000	0.6663	0.3559

Values in italics are with a significance level at *p* < 0.05

The highest pH was observed at the beginning of experiment and showed a very rapid decrease during the first 25 days. After that, the values stabilized for each column at the level of ≈ 8.4. There is seen an influence of CEs addition for the pH: 13.15, 13.17, and 13.20 for C1, C2, and C3, respectively. Whereas, the initial pH of C0 was 7.39. It is also confirmed by a significant relationship with TDS and EC for C1–C3 (Tables 6, 7, and 8).

**Table 6:** Pearson correlation coefficients between physical, chemical parameter leaching, and flow for C1

	Q	TDS	pH	EC	P-PO_4_	Average	SD
Q	1.000000					0.0218	0.0085
TDS	*0.700800*	1.000000				539.0238	217.8305
pH	*0.650058*	*0.435602*	1.000000			9.1686	1.0130
EC	*0.802724*	*0.944310*	*0.647127*	1.000000		1.1690	0.8153
P-PO_4_	*− 0.344969*	− 0.054662	*− 0.548850*	− 0.230717	1.000000	0.3837	0.2424

Values in italics are with a significance level at *p* < 0.05

**Table 7:** Pearson correlation coefficients between physical, chemical parameter leaching, and flow for C2

	Q	TDS	pH	EC	P-PO_4_	Average	SD
Q	1.000000					0.0216	0.0060
TDS	*0.399707*	1.000000				545.0714	239.6898
pH	*0.439026*	*0.603296*	1.000000			9.3443	1.5587
EC	*0.430845*	*0.991449*	*0.564571*	1.000000		1.1324	0.8910
P-PO_4_	− 0.263697	*− 0.338126*	*− 0.581336*	− 0.300810	1.000000	0.2942	0.2328

Values in italics are with a significance level at *p* < 0.05

**Table 8:** Pearson correlation coefficients between physical, chemical parameter leaching, and flow for C3

	Q	TDS	pH	EC	P-PO_4_	Average	SD
Q	1.000000					0.0217	0.0068
TDS	*0.769481*	1.000000				583.9286	291.0606
pH	*0.538850*	*0.718759*	1.000000			9.7221	1.8050
EC	*0.787559*	*0.985674*	*0.649890*	1.000000		1.2507	1.0365
P-PO_4_	*− 0.386459*	*− 0.469399*	*− 0.741555*	*− 0.425375*	1.000000	0.0508	0.0472

Values in italics are with a significance level at *p* < 0.05

In the case of C0, it is observed a washout of P-PO_4_ from sand thus a higher concentration at the beginning decreasing from 1.875 to 0.547 mg L^−1^. For C1–C3, we observed an influence of CEs addition. The extreme values were 0.000–0.788 mg L^−1^, 0.000–0.718 mg L^−1^, and 0.000–0.140 mg L^−1^ for C1, C2, and C3, respectively. The mass of CEs significantly influence of EC, TDS, and pH because only for C3, each of the Pearson coefficients are significant (Tables 6, 7, and 8).

**Fig. 4:**
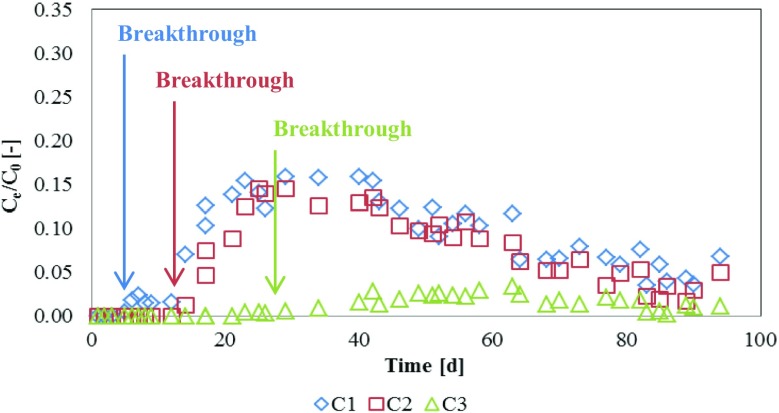
The level of P-PO_4_ saturation (*C*_e_/*C*_0_) in C1–C3

Data obtained from the outflow of C1–C3 columns were evaluated with respect to P-PO_4_ breakthrough (Fig. 4). The breakthrough was appeared after 6 (C1), 14 (C2), and 25 (C3) days after opening. These correspond to volume of 182, 385 and 771 mL that passed through the 1st, 2nd, and 3rd column, respectively.

**Fig. 5:**
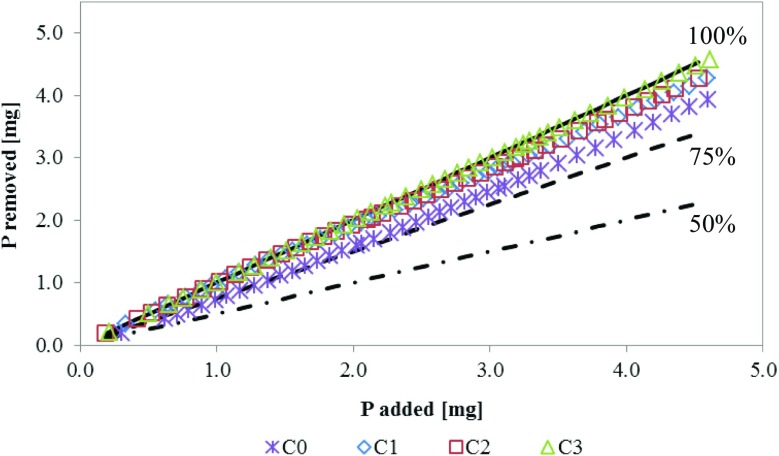
P-PO_4_ removed as a function of P-PO_4_ added

The total column retained loads of P-PO_4_ obtained during the experiment were 3.931, 4.267, 4.277, and 4.573 mg P-PO_4_ for C0, C1, C2, and C3, respectively. That correspond to percentage removal ratio of 97.32 (C0); 98.33 (C1); 98.68 (C2); and 99.76 (C3). The sum of each column load is presented in Fig. 5. Converting the retained loads into unit sorption obtained by CEs received an inverse result: 10.668, 4.277, and 2.286 mg P-PO_4_ g^−1^ for C1, C2, and C3, respectively. Because of low unit sorption obtained from C0 (0.098 mg g^−1^), the influence of sand was not included with sorption obtained from C1 to C3.

Also, the data obtained for C0–C3 were analyzed by analysis of variance (ANOVA) and Tukey’s test at a significance level of 0.05. Performed analysis showed that there is a significant difference between columns.

## Discussion

### Sorption Studies

The kinetic and equilibrium sorption studies with thermal and chemical modification eggshells were a subject of many studies (Köse and Kıvanç 2011, Guo et al. 2017, Mezenner and Bensmaili 2009, Zhang et al. 2018) and fitted both to different isotherm models. In contrary to this research, Köse and Kıvanç (2011) obtained for eggshells calcined in 800 °C better fit to the Freundlich (*K* = 23.02 mgP-PO_4_ g^−1^) isotherm model than to Langmuir and the adsorption is defined as a physical. On the other hand, Guo et al. (2017) had a better fit to the Langmuir isotherm for parent eggshells with *S*_max_ values ranged from 0.26 to 0.57 mgP g^−1^. The obtained by them kinetic studies follow pseudo second-order model which indicates a chemisorption as a P removal mechanism. Also, Koumanova et al. (2002) obtained good suitability to the Langmuir model that confirmed the chemisorption.

Better sorption results were noted for chemically modified eggshells by Al (Guo et al. 2017, Zhang et al. 2018) and Fe (Mezenner and Bensmaili 2009). The Al-modified eggshells were characterized by good fit both to Freundlich (Guo et al. 2017, Zhang et al. 2018) and the Langmuir models (Guo et al. 2017). Guo et al. (2017) obtained the *q*_max_ values ranged from 3.58 to 6.23 mgP g^−1^ (from 11 to 17 times higher than for parent eggshell examined in the same study). Also, the kinetic studies confirmed that sorption process runs faster in the case of Al-modified eggshells and the equilibrium was reached after 600 and 90 min for patent and modified eggshells, respectively. On the other hand, Zhang et al. (2018) for Al-modified eggshells stated good suitability to the Freundlich model. The pseudo second-order kinetic model describes the experimental data well and implies that adsorption is controlled by chemisorption. Similar observation was reported by Mezenner and Bensmaili (2009) for Fe-modified eggshell. At lower temperature (20 and 25 °C), the isotherm is a better fit to the Freundlich model, and at higher (35 and 45 °C) to the Langmuir one, with *S*_max_ (12.51–14.49 mg g^−1^). The analyzed RM characterized by well fit to pseudo second kinetic order model.

Observed high pH (Fig. 2) values are a consequence of content of Ca in material (Table 1). According to Yang et al. (2013), phosphate equilibrium in aqueous solution is pH dependent. The level of pH higher than 12 favors to promote ${\mathrm{PO}}_4^{3-}$ species (Zhang et al. 2018). During calcination process at 900 °C, CaCO_3_ was converted to CaO which is highly reactive to phosphorus. Under alkaline conditions, direct precipitation Ca_3_(PO_4_)_2_ and Ca_5_(PO_4_)_3_OH are achieved following Eqs. 6 and 7 (Chen et al. 2013):

6 $$ 5{\mathrm{Ca}}^{2+}+3{\mathrm{H}\mathrm{PO}}_4^{2-}+4{\mathrm{OH}}^{-}={\mathrm{Ca}}_5\left({\mathrm{PO}}_4\right)3\mathrm{OH}\downarrow +3{\mathrm{H}}_2\mathrm{O} $$

7 $$ 3{\mathrm{Ca}}^{2+}+2{\mathrm{H}\mathrm{PO}}_4^{2-}+2{\mathrm{OH}}^{-}=\mathrm{Ca}3\left({\mathrm{PO}}_4\right)2\downarrow +2{\mathrm{H}}_2\mathrm{O} $$

Haghseresht et al. (2009) confirmed that the sorption capacity increased when the concentrations of ${\mathrm{H}}_2{\mathrm{PO}}_4^{-},{\mathrm{H}\mathrm{PO}}_4^{2-},$ and ${\mathrm{PO}}_4^{3-}$ species increased, indicating that calcium ions had a greater affinity for the dihydrogen phosphate, hydrogen phosphate, and phosphate. Also, Chen et al. (2014) claimed that at high pH in aqueous solution, a chemical precipitation mechanism may be the predominant process.

### Column Experiment—Breakthrough and Saturation Estimation

There is lack of column experiment carried out with CEs used to remove P from synthetic solution or surface water/wastewater. For these reasons, comparing obtained data with literature is quite difficult. To our knowledge, the only research made with raw eggshell as a reactive media in a column experiment was carried out by Vijayaraghavan et al. (2005) and regarded removal of copper.

The way of estimating the longevity of RMs and their reactivity in the case of treated media are breakthrough curves. On the ideal curve, two phases should be observed: 1st until the breakthrough appears and 2nd when the saturation of RM is observed. In this study, breakthrough was defined to be reached when the ratio between effluent and influent P-PO_4_ concentrations equal > 0.0. However, this a conventional indicator and Herrmann et al. (2014) as a breakthrough indicated the ratio of ≥ 0.08 and ≥ 0.02 for initial P concentrations of 12 and 50 mg L^−1^, respectively. The saturation is a time consuming process and appears when the effluent concentration equals the influent one. Most researchers achieved only the breakthrough point, not the saturation level of material (Nilsson et al. 2013, Kang et al. 2017). The shape of *C*_e_/*C*_o_ curves obtained in this study differs from those obtained by, e.g., Nilsson et al. (2013) and Kang et al. (2017). Nilsson et al. (2013) for Polonite® and Sorbulite during 90 days experiment reach ≈ 0.20 and ≈ 0.25 with a sorption of 1.14 and 2.64 mgP-PO_4_ g^−1^ for Polonite® and Sorbulite, respectively. Kang et al. (2017) for non-treated (NT-CCG) and thermal-treated in 700 °C crushed concrete granules (700TT-CCG) columns with sand amendments during 300 h experiment did not observe breakthroughs. However, for sand column, the curves increased sharply, reaching 0.8 during 1 h. Because of short working time of column experiment (300 h), the sorption was low and equaled 0.06949 and 0.07134 mg-${PO}_4^{3-}$·g^−1^ for NT-CCG and 700TT-CCG, respectively.

The breakthrough and saturation level often depend on such factors as mass of RM (bed height), initial concentration, and retention time; however, the most important is a flow rate. The shape of *C*_e_/*C*_o_ curves from this study corresponds to these obtained by Adam et al. (2005) because of flow rate differences between columns that influence on retention time. Adam et al. (2005) for boxes experiment with high inlet P concentration (15 ppm) and load rate (2.5–5.0 L day^−1^) reached 90% saturation after 150 days of operation, while boxes with low hydraulic load (1.25 L day^−1^) reach 70–99% saturation after 1.5 years. Similar observation reported Vijayaraghavan et al. (2005). Increasing flow rate from 5 to 20 mL min^−1^ shortens the breakthrough time from 3.9 to 1.3 h and exhaustion (saturation) time from 19.8 to 6.8 h. Both those studies confirmed that increasing of loading rate leads to a faster breakthrough. In contrary, Herrmann et al. (2013) indicated the negative influence of loading rate on P binding capacity with Filtralite P®: with increasing load rate, the breakthrough and saturation levels are also increasing. Opposite to other study results, they explained by an increased washout of P precipitates that occur with contact of filter materials with water. The calcium ions probably hydrolyze through the watering of Ca-containing compounds in the material, reacting with phosphate and precipitate. Then, the precipitates are physically or mechanically retained in the filter.

### Increasing Reduction vs. Decreasing Sorption

Observed decreasing unit sorption (mg g^−1^) with increasing removal of P (%) with simultaneous decrease of RM mass to volume ratio was previously observed by Yeddou and Bensmaili (2007), Chen et al. (2013), and Bus and Karczmarczyk (2017). Yeddou and Bensmaili (2007) explained the effect of increasing the mass to volume ratio from 1 to 5 g L^−1^ by aggregating, overlapping, and overcrowding particles of the higher dose of material. This resulted in a decrease of the availability of the surface area as well as decrease of the sorption capacity. Also, Chen et al. (2013) noted P sorption capacity ranged from 0.119 to 0.910 mg g^−1^ and decreasing of the removal ratio from 99.17 to 91.00%. They claim that this happens due to the ratio between P ion and the available binding sites. At low initial P concentration, the availability of binding sites is relatively higher. The suggested removal ratio exceeded 90% is considered that the RM can provide a significant P removal for wastewater in a wide concentration range.

### Practical Implications

The recommended hydraulic loading rate (HLR) for on-site wastewater treatment ranges from 30 to 40 L m^−2^ day^−1^ (EPA/625/R-00/008 2002). In this study, the average HLR equaled 203 and it is from 5 to 7 times higher than recommended. If the recommended HLR value was used, the experiment would be overlarge. Also, Vijayaraghavan et al. (2005) used the small-scale columns for obtaining the sorption capacity of eggshells: 35 cm in height (the bed height ranged from 15 to 25 cm) and 2 cm in diameter with a flow rate of 5–20 mL min^−1^. It is expected that decreasing the HLR to recommended value increases the sorption obtained from the experiment (Vijayaraghavan et al. 2005, Adam et al. 2005) because of longer contact time, longer time to obtain the saturation level, and incidental drying of filter that also may influence positively on sorption. What is more, the sand was used as a filter protection from fast clogging (because of used fine fraction of CEs) and extremely alkaline leakages from the filter (noted pH equals even 13.19; see isotherm studies). Because of difficulties with obtaining the CEs on an industrial scale, the addition of CEs ranged from 1 to 5% of total mass of column and seems to be justified.

Even after the 95 days of experiment, the outlet P concentration was equaled to 0.387, 0.281, and 0.063 mg L^−1^ that corresponds to removal of 93, 95, and 99% for C1, C2, and C3, respectively. These values are still lower than required effluent concentration from on-site wastewater treatment plant located at urban areas in Poland (2.0 mg L^−1^ for PE > 10,000 and 1.0 mg L^−1^ for PE > 100,000; Dz.U./2014/1800), small-scale wastewater facilities (< 25 PE) in Sweden (3.0 and 1.0 mg L^−1^ for common and highly sensitive receiving waters, respectively, Vidal et al. 2018), or small systems in Norway (1.0 mg L^−1^; Heistad et al. 2006).

The CEs may be used as a support of septic tank system (STS) in increasing the P removal efficiency. Designing the STS with a dimension of 60 m length, 0.5 m width, and 0.10 m high as a P filter, the mixture of sand and CEs can be used. For this, 3 m^3^ of RMF for filling up the filter is recommended. The dry density of sand used in this study was 1625.48 kg m^3^. Assuming the addition of CEs of 1.0, 2.5, and 5.0%, the mass of CEs is as follows: 49, 122, and 244 kg.

## Conclusions

The calcined eggshells are characterized by high sorption properties and fast and rapid reaction with phosphates. Obtained sorption from both batches and column experiment presents promising values. The modeled maximum sorption capacity by the Langmuir isotherm model was 72.87 mgP g^−1^. The contact time of 5 min was sufficient to remove P-PO_4_ from solution completely.

The amendment of CEs ranged from 1.0 to 5.0% of column filling seems to be sufficient for phosphates removal. The sorption unit obtained during 95 days of column experiment was 10.668, 4.277, and 2.286 mg P-PO_4_ g^−1^ for 1.0, 2.5, and 5.0%, respectively. During this time, the breakthrough for C1–C3 was reached; however, the saturation of the CEs was not observed. The obtained results in this study may be used as a groundwork for perspective usage of CEs at on-site wastewater treatment plants.
